# Increased risk of chronic fatigue and hair loss following COVID-19 in individuals with hypohidrotic ectodermal dysplasia

**DOI:** 10.1186/s13023-021-02011-z

**Published:** 2021-09-03

**Authors:** Verena Hennig, Wolfgang Schuh, Antje Neubert, Dirk Mielenz, Hans-Martin Jäck, Holm Schneider

**Affiliations:** 1 Department of Pediatrics, University Hospital Erlangen, University of Erlangen-Nürnberg, Loschgestr. 15, 91054 Erlangen, Germany; 2 Center for Ectodermal Dysplasias, University Hospital Erlangen, University of Erlangen-Nürnberg, Erlangen, Germany; 3 Division of Molecular Immunology, University Hospital Erlangen, University of Erlangen-Nürnberg, Erlangen, Germany

**Keywords:** Hypohidrotic ectodermal dysplasia, SARS-CoV-2, COVID-19, Chronic fatigue, Hair loss

## Abstract

**Background:**

Hypohidrotic ectodermal dysplasia (HED) is a group of genodermatoses in which deficient ectodysplasin A signalling leads to maldevelopment of skin appendages, various eccrine glands, and teeth. Individuals with HED often have disrupted epithelial barriers and, therefore, were suspected to be more susceptible to coronavirus infection.

**Methods:**

56 households with at least one member who had coronavirus disease of 2019 (COVID-19) were enrolled in a longitudinal study to compare the course of illness, immune responses, and long-term consequences of severe acute respiratory syndrome-coronavirus 2 (SARS-CoV-2) infection in HED patients (n = 15, age 9–52 years) and control subjects of the same age group (n = 149).

**Results:**

In 14 HED patients, mild or moderate typical COVID-19 symptoms were observed that lasted for 4–45 days. Fever during the first days sometimes required external cooling measures. The course of COVID-19 was similar to that in control subjects if patients developed antibodies blocking the SARS-CoV-2 spike protein. Five out of six HED patients with completely abrogated ectodysplasin A signalling (83%) suffered from chronic, in two cases very severe fatigue following COVID-19, while only 25% of HED patients with residual activity of this pathway and 21% of control subjects recovering from COVID-19 experienced postinfectious fatigue. Hair loss after COVID-19 was also more frequent among HED patients (64%) than in the control group (13%).

**Conclusions:**

HED appears to be associated with an increased risk of long-term consequences of a SARS-CoV-2 infection. Preventive vaccination against COVID-19 should be recommended for individuals affected by this rare genetic disorder.

**Supplementary Information:**

The online version contains supplementary material available at 10.1186/s13023-021-02011-z.

## Introduction

Genetic conditions may play a role in the susceptibility to COVID-19, its progression and outcome [1, 2]. Hypohidrotic ectodermal dysplasia (HED) is a group of rare genetic disorders affecting the development of structures derived from the embryonic ectoderm, such as hair, sweat and sebaceous glands, lacrimal and Meibomian glands in the eyelids, airway submucosal glands, and teeth [3]. These disorders are caused by pathogenic variants of genes involved in the ectodysplasin A signalling pathway [4, 5]. Individuals with HED have very dry mucous membranes in the upper respiratory tract and the eyes, leading to disrupted epithelial barriers [6, 7] that could facilitate the entry of severe acute respiratory syndrome-coronavirus type 2 (SARS-CoV-2) into the body. Deficient ectodysplasin A signalling might also limit antiviral immune responses [4]. Furthermore, the lack of sweat glands is associated with a high risk of life-threatening hyperthermia during febrile illness, particularly in early childhood [8, 9]. Based on this knowledge, HED patients were suspected to be more prone to SARS-CoV-2 infection and to a severe course of COVID-19 irrespective of their age.

Children become less frequently infected by SARS-CoV-2 and usually have less intense symptoms than adults with COVID-19 [10, 11]. Chinese researchers who investigated household contacts of a larger group of COVID-19 patients reported a secondary attack rate of 4% for children compared with 17.1% for adults [12]. Studies of complete households also appeared to be an appropriate method to assess the susceptibility to SARS-CoV-2 infection in paediatric and adult HED patients and both the severity of disease and its long-term consequences in this subpopulation.

During the first months of the COVID-19 pandemic, our expert centre, therefore, initiated a longitudinal study to investigate the course of illness, immune responses, complications and late-onset symptoms of COVID-19, such as postinfectious fatigue, in complete households with children or adolescents.

## Subjects and methods

### Study design and recruitment of patients

Seven male and eight female individuals between 9 and 52 years of age with genetically confirmed HED were enrolled in a longitudinal observational study of 56 complete households with at least one member who had COVID-19 to investigate the spreading and course of illness, immune responses, and long-term consequences of SARS-CoV-2 infection (ClinicalTrials.gov identifier: NCT04741412). A total of 149 subjects of the same age group but from households without a family history of HED served as controls. The study was approved by the ethics committee of the University Erlangen-Nürnberg and conducted in accordance with the principles of the declaration of Helsinki. All individuals or their legal guardians provided written informed consent to participate.

The protocol included the collection of relevant clinical data from medical records, reverse transcription-polymerase chain reaction (RT-PCR) analysis of material from respiratory tract swabs, repeated blood sampling and analysis, clinical examinations, and structured interviews. SARS-CoV-2 infection was diagnosed based on exposure history, clinical manifestation, and a positive RT-PCR result. In the absence of specific PCR results, a combination of COVID-19 symptoms or daily contact to an affected household member and subsequent detectability of antibodies against the SARS-CoV-2 spike protein and/or nucleocapsid at two or more points in time led to the retrospective diagnosis of a SARS-CoV-2 infection. Patient-reported long-term consequences of COVID-19 were registered using a specific questionnaire and followed up by repeated phone interviews and gathering of all relevant medical documents. Reports of severe hair loss were confirmed by clinical examination. The final data analysis was conducted in May 2021.

Subjects were included only if one or more household members had been registered as patient(s) in databases of the University Hospital Erlangen and if at least one person in the household was younger than 18 years of age. Exclusion criteria were missing informed consent of one or more household members and language barriers to communication that would prevent informed consent.

### PCR analysis

RT-PCR to detect SARS-CoV-2 was performed using the qualitative Cobas SARS‐CoV‐2 dual‐target RT‐PCR assay (SARS‐CoV‐2 specific target 1: ORF1/a region; pan‐Sarbecovirus‐specific target 2: envelope E region) from Roche Diagnostics (Mannheim, Germany). Material from respiratory tract swabs was investigated. This assay, running on an automated PCR system (Cobas 6800; Roche Diagnostics, Mannheim, Germany), displays cycle threshold (Ct) values for both viral target sequences and the internal control. According to the manufacturer's evaluation, the detection limit for SARS‐CoV‐2 RNA in respiratory tract swabs is 0.009 tissue culture infectious dose 50% (TCID50)/mL for target 1 and 0.003 TCID50/mL for target 2.

### Detection of specific antibodies

Patients’ sera were screened for anti-SARS-CoV-2 antibodies using the NADAL COVID-19 IgG/IgM test (nal von minden GmbH, Moers, Germany) or the Anti-SARS-CoV-2 ELISA (Euroimmun, Lübeck, Germany) according to the manufacturers’ instructions.

Flow cytometry-based detection of antibodies (IgA, IgM, and IgG) against the SARS-CoV-2 spike protein were conducted as described previously [13, 14]. In brief: A plasmid encoding SARS-CoV-2 spike (QHD43416.1, aa1–1273, position 21,580–25,400 from GenBank NC_045512) was co-transfected together with a green fluorescent protein (GFP)-encoding plasmid into HEK293T cells. Two days later the cells were incubated with serum samples from patients (1:100 dilution), followed by staining with a secondary antibody mixture of PE-conjugated anti-human IgA (Southern Biotech, Birmingham, U.S.), AF647-conjugated anti-human IgG (Southern Biotech) and DyLight405-conjugated anti-human IgM (Jackson ImmunoResearch, Ely, U.K.) antibodies. Stained cell populations were analyzed using a Gallios flow cytometer (Beckman-Coulter). The antibody TRES 224 [15] recognizing the SARS-CoV-2 spike protein served as positive control.

Specific antibodies blocking the spike protein of SARS-CoV-2 were identified using a cell-based SARS-CoV-2 spike surrogate blocking assay, details of which have been published in a separate manuscript by our group [14].

Quantification of IgG specific for the SARS-CoV-2 nucleocapsid or the spike S1 protein was performed by SYNLAB International GmbH (Weiden, Germany).

### Assessment of pulmonary function

Pulmonary function tests included assessments of vital capacity (VC In), forced expiratory volume over 1 s (FEV1), peak flow (PEF), and maximal expiratory flow at 75% of the forced vital capacity (MEF75). The results were interpreted by an experienced paediatric pneumologist.

### Statistical analysis

Datasets were analyzed with unpaired t-tests using the GraphPad Prism software 7 (GraphPad Software Inc., La Jolla, USA).

## Results

In our cohort of individuals with HED, nine of the 15 subjects acquired the SARS-CoV-2 infection within their household from affected family members, the other six patients from external contacts. COVID-19 symptoms (fever, headache, myalgia, sore throat, hoarseness, cough, chest tightness, neurological and gastrointestinal symptoms) were mild to moderate and did not differ from those of control subjects. Fever during the first days, however, required external cooling measures in five adults with the full-blown phenotype of HED, although none of these patients had to be hospitalized. PCR testing for SARS-CoV-2 was performed 1–11 days after onset of symptoms, except for two women who were quarantined based on typical clinical findings in March 2020 (at the beginning of the first wave of COVID-19 in Germany) and the asymptomatic son of one of them. PCR results confirmed the clinical diagnosis of COVID-19 in each case. Demographic and genetic characteristics of the HED patients investigated are summarized in Table 1.

**Table 1:** Characteristics of the 15 subjects with hypohidrotic ectodermal dysplasia (HED) investigated in this study

Subject	Sex	Age	HED-related genotype		Zygosity	Ectodysplasin A	Blood	PCR test for	Duration of	IgG against	Postinfectious
	(m/f)	(years)	Gene	Pathogenic variant		Signalling	group	SARS-CoV-2	COVID-19	SARS-CoV-2	fatigue
ED-A1	m	49	*EDA*	c.1091T > G (p.M364R)	Hemizygous	Abrogated	A Rh +	pos. (03/2020)	22 days	Yes	Moderate
ED-A2	f	27	*EDAR*	c.486delC	Homozygous	Abrogated	B Rh +	n.d. (03/2020)	8 days	Yes	Severe
ED-A3	m	20	*EDA*	c.896G > C (p.G299A)	Hemizygous	Abrogated	0 Rh +	pos. (11/2020)	13 days	Yes	None
ED-A4	m	46	*EDA*	del exons 4–9	Hemizygous	Abrogated	0 Rh +	pos. (12/2020)	23 days	Yes	Severe
ED-Y1	m	15	*EDA*	c.896G > C (p.G299A)	Hemizygous	Abrogated	0 Rh +	pos. (10/2020)	45 days	Yes	Moderate
ED-Y2	m	16	*EDA*	c.477C > T (p.R153C)	Hemizygous	Abrogated	n.d	pos. (11/2020)	12 days	Yes	Extreme
ED-C1	m	9	*EDA*	c.45_49del5	Hemizygous	Abrogated	n.d	n.d. (03/2020)	**no symptoms**	Yes	None
ED-A5	f	37	*EDA*	c.466C > T (p.R156C)	heterozygous	residual activity	0 Rh +	pos. (03/2020)	13 days	Yes	None
ED-A6	f	40	*EDA*	c.45_49del5	Heterozygous	Residual activity	0 Rh +	n.d. (03/2020)	27 days	Yes	None
ED-A7	f	19	*EDA*	c.896G > C (p.G299A)	Heterozygous	Residual activity	0 Rh-	pos. (10/2020)	3 days	yes	none
ED-A8	m	22	*EDA*	c.769G > C (p.G257R)	Hemizygous	Residual activity	0 Rh-	pos. (10/2020)	7 days	Yes	None
ED-A9	f	42	*EDA*	c.896G > C (p.G299A)	Heterozygous	Residual activity	0 Rh-	pos. (10/2020)	21 days	Yes	Mild
ED-A10	f	46	*EDA*	c.477C > T (p.R153C)	Heterozygous	Residual activity	A Rh +	pos. (11/2020)	4 days	Yes	None
ED-A11	f	52	*EDA*	c.896G > C (p.G299A)	Heterozygous	Residual activity	0 Rh +	pos. (11/2020)	20 days	Yes	Moderate
ED-Y3	f	16	*EDA*	c.1091T > G (p.M364R)	Heterozygous	Residual activity	A Rh +	pos. (03/2020)	7 days	Yes	None

Bold highlights the only patient without clinical symptoms

m, male; f, female; n.d., not determined; pos.**,** positive test result

### Immune responses to SARS-CoV-2

**Fig. 1:**
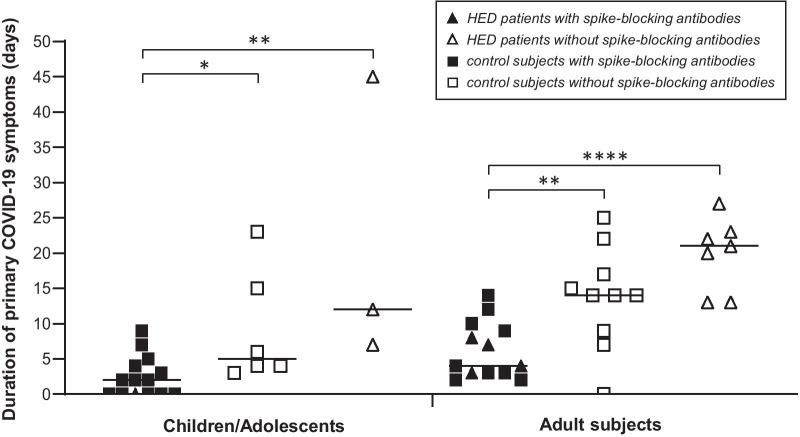
Duration of COVID-19 in HED patients with or without antibodies that block binding of the SARS-CoV-2 spike protein to its receptor ACE2. Sera from individuals with HED and SARS-CoV-2 infection (triangles, n = 15, age 9–52 years) and from control subjects of the same age group who had been exposed to SARS-CoV-2 (squares, n = 38) were investigated for specific spike-blocking antibodies against this virus. If such antibodies were present, the duration of primary COVID-19 symptoms (fever, head and body ache, diarrhea, exhaustion, cough, shortness of breath, and chest tightness) was similar to that of control subjects. In the absence of spike-blocking antibodies against SARS-CoV-2, however, the course of COVID-19 was often remarkably long

IgG against SARS-CoV-2 (detected by ELISA and by flow cytometry) developed within a few weeks and remained detectable in the blood for at least four months in 14 of 15 HED patients (93.3%) and in 95.9% of the control subjects (data not shown). All children and most of the adults with SARS-CoV-2 infection also produced IgA against this virus. Cellular immunity was not studied systematically. In the sera of five HED patients (four adults, one child), we found specific antibodies blocking the interaction of the spike protein of SARS-CoV-2 with recombinant human angiotensin-converting enzyme 2 (ACE2) (Fig. 1 and Additional file 1: Figure S1). COVID-19 symptoms of these patients had lasted for a maximum of 8 days, well within the range of the control group, while the duration of primary COVID-19 symptoms was much longer in subjects without such spike-blocking antibodies (on average 21.3 days in children/adolescents and 19.9 days in adults affected by HED, compared with 9.2 and 13.7 days in control children/adolescents and control adults, respectively).

In the majority of subjects investigated over 12 and more months, SARS-CoV-2 antibodies were still detectable by ELISA one year after the coronavirus infection: IgG against the nucleocapsid of SARS-CoV-2 in 70.6% of the adults and 77.8% of children/adolescents, IgG against the SARS-CoV-2 spike protein in 58.8% of the adults and 77.8% of children/adolescents with or without HED, although the serum concentrations of both types of IgG showed a tendency to be lower in HED patients than in healthy control subjects (Table 2).

**Table 2:** Detectability of antibodies against SARS-CoV-2 one year after infection

	Adults (age > 17 years)	Children/adolescents
*HED subjects (n = 6)*		
Subjects with antibodies against the SARS-CoV-2 nucleocapsid	3/4	1/2
Subjects with antibodies against the SARS-CoV-2 spike S1 protein	3/4	1/2
Average concentration (BAU/ml)	88.17	57.60
Standard deviation	32.43	n. a
*Control subjects (n = 20)*		
Subjects with antibodies against the SARS-CoV-2 nucleocapsid	9/13	6/7
Subjects with antibodies against the SARS-CoV-2 spike S1 protein	7/13	6/7
Average concentration (BAU/ml)	170.31	160.45
Standard deviation	118.44	137.41

### Postinfectious fatigue

**Fig. 2:**
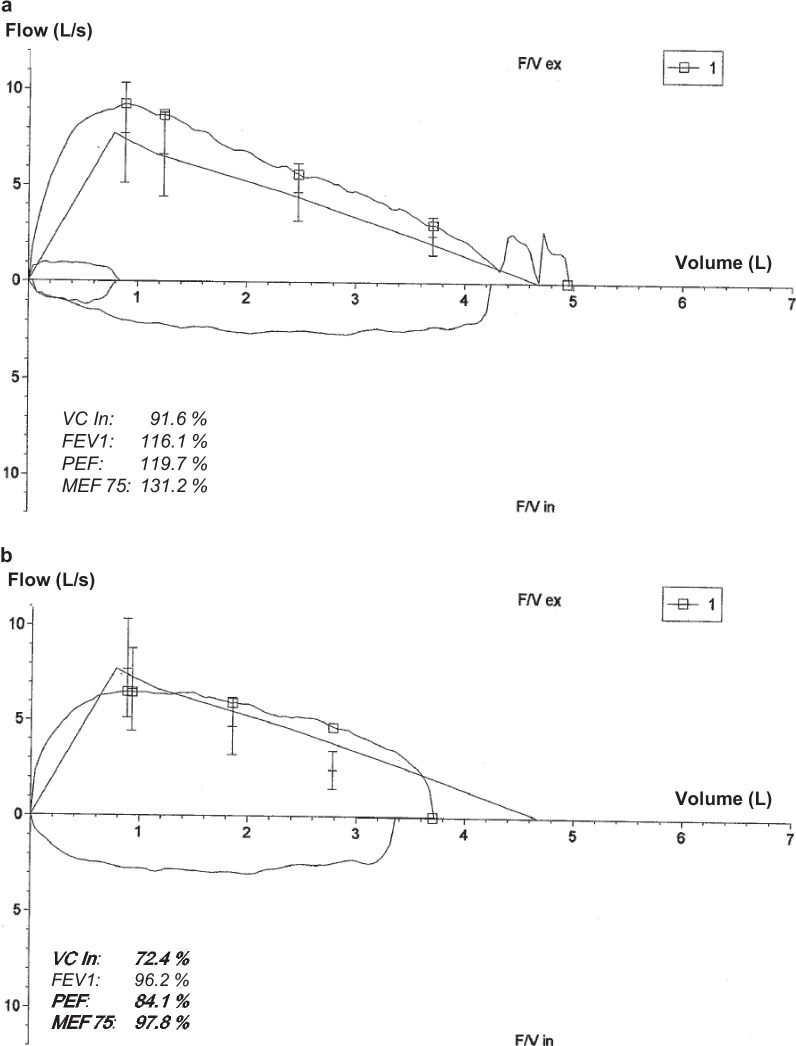
Results of pulmonary function tests of a 16-year-old subject with HED before SARS-CoV-2 infection (**a**) and 7 days after recovery from primary COVID-19 symptoms (**b**). This boy also experienced extreme postinfectious fatigue

Subject ED-Y2, a 16-year-old male adolescent with HED and active baseball player who had regularly taken part in team competitions in the second-highest German national league, experienced COVID-19 symptoms including headache, fever, myalgia, and cough for a total of 11 days. The patient recovered fully and started practicing sports again. Four days after the end of his quarantine, he suddenly developed very severe fatigue: He complained about a constant lack of energy, became unable to leave the bed for more than 30–40 min per day, had difficulties staying awake when eating, and could not take part in school lessons online. Neurological or cardiovascular disorders and depression were ruled out by medical specialists. A pulmonary function test indicated restrictive lung disease (Fig. 2) that did, however, not explain the extreme fatigue. This condition persisted for 12 weeks, before minor improvement was noticed. Postinfectious fatigue excluding a return to sports has not resolved up to now, six months after the infection, while pulmonary restrictions and cognitive disturbances have almost disappeared. After a long-term complex rehabilitation, the patient hopes to be able to return to school in the following weeks.

Four of the five other HED patients who are hemi- or homozygous for null mutations in the disease-causing gene (three men, one woman; Table 1) complained about prolonged, often severe fatigue following COVID-19 that had a major impact on their capacity to work. This was accompanied by shortness of breath in two subjects. Chronic fatigue was clearly more frequent among individuals with completely abrogated ectodysplasin A signalling (83%) than in HED patients with residual activity of the ectodysplasin-NF-kB pathway (2/8; 25%). The latter subgroup experienced postinfectious fatigue at a similar frequency as control subjects recovering from COVID-19 (23/108; 21%).

### Postinfectious hair loss and other issues

**Fig. 3:**
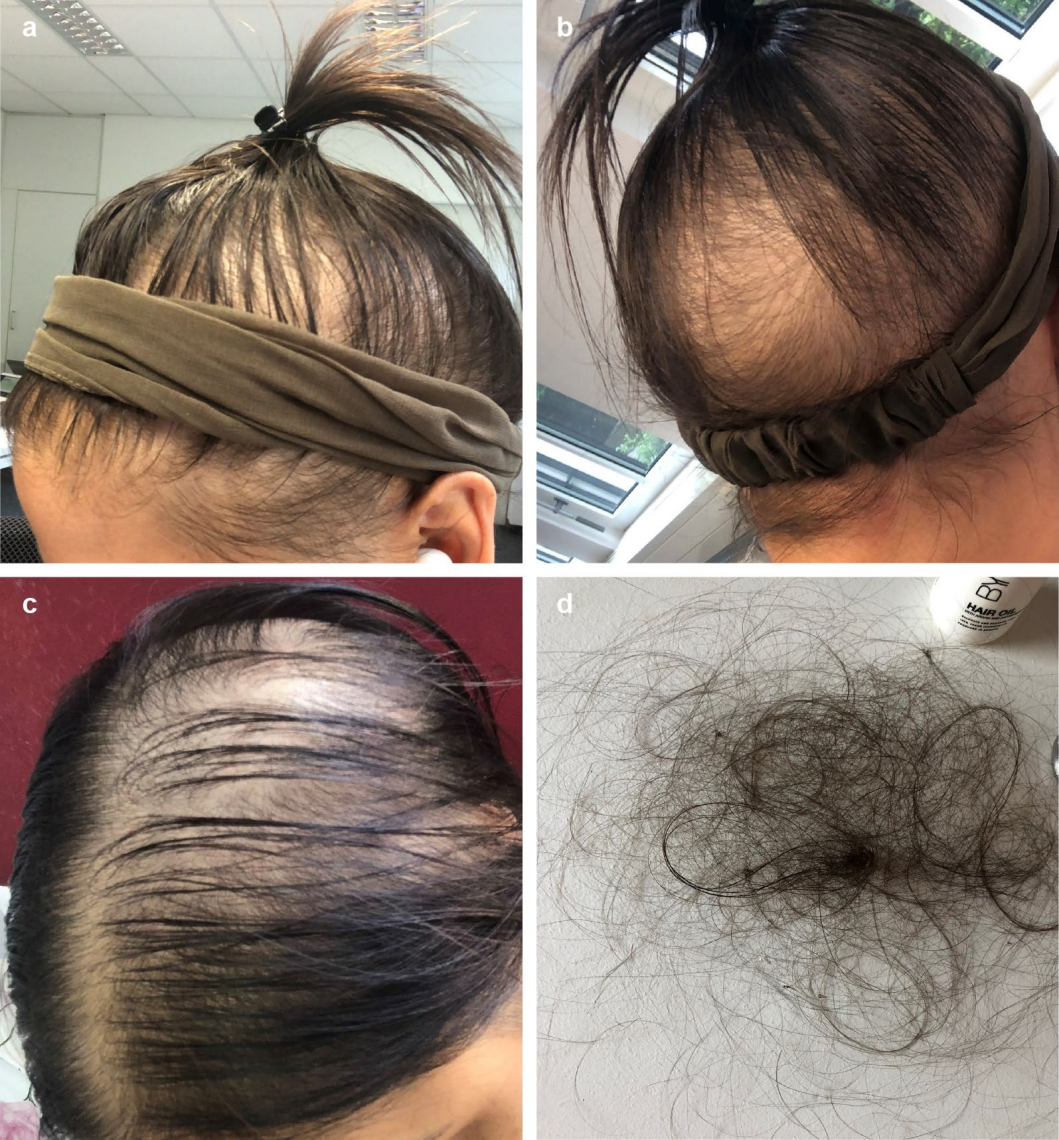
Hair loss ten weeks after recovery from COVID-19 in a 27-year-old woman with autosomal recessive HED. **a**, **b** Progressive reduction in the number of hairs, especially in the central, frontal, and parietal scalp regions. **c** Diffuse thinning of the upper biparietal and vertex regions and preservation of the anterior hair implantation line. **d** Clumps of hair shed after slight traction on the scalp hair during daily brushing

Eight adult HED patients (six women and two men) reported noticeable hair loss subsequent to COVID-19. Again, this phenomenon affected HED patients (8/14; 64%) more often than control subjects (14/108; 13%) and was a relevant health issue, particularly for female patients (Fig. 3). Hair loss started suddenly with clumps of hair falling out while combing or brushing (Fig. 3) approximately two months after the onset of COVID-19 symptoms and lasted for up to six months.

Phone interviews and clinical examinations also highlighted recurrent nail issues affecting transiently both finger- and toenails, such as brittle nails that frequently cracked or split, but no loss of entire nails was reported.

## Discussion

Although the deficient development of various eccrine glands in HED patients entails recurrent respiratory infections, atrophic rhinitis, and keratoconjunctivitis sicca [6, 16], neither the course of COVID-19 nor humoral immune responses to SARS-CoV-2 seem to differ considerably from those in control subjects. Our data further suggest that serum antibodies blocking the binding of SARS-CoV-2 spike protein to ACE2 are a factor with relevant influence on the duration of COVID-19 both in HED patients and control subjects. Remarkable and unexpected differences, however, were observed concerning postinfectious fatigue, a chronic illness with constitutional and neurocognitive symptoms persisting long after clearance of the initial infection [17]. Chronic fatigue is a multifactorial and poorly understood consequence of a variety of predominantly viral infections. Coxiella burnetii, Epstein-Barr virus, influenza virus, and other infectious agents have been hypothesized to cause persistent dysregulation of inflammatory response and metabolic pathways, most likely related to a miscommunication in cytokine networks [17, 18]. There is an increasing number of individuals who suffer from post-acute COVID-19, an even more complex disease entity characterized by persistent symptoms and/or delayed or long-term complications beyond 4 weeks from the onset of symptoms [19, 20], including anosmia, muscular weakness, cognitive and sleep disturbances, chest pain, palpitations, and hair loss. A systematic meta-analysis of cohort studies [21] revealed prolonged effects after acute COVID-19 in 80% of the study population aged 17–87 years. Persistent respiratory symptoms, rather independent of the severity of initial lung involvement [22], have often been recorded and follow-up of large groups of patients indicated breathlessness to be one of the most frequent postinfectious health issues [23, 24]. This was also evident among our HED patients. While pulmonary problems had been expected in patients with a full-blown phenotype of HED, the striking difference in the frequency and severity of chronic fatigue was surprising. In contrast to the general population, where “long haulers” are predominantly female [25], male subjects with HED were affected more often by chronic fatigue than women with this rare disorder. This is explained by the large proportion of X-linked HED in our cohort (93.3%).

Hair loss, the second issue more frequent among HED subjects than control subjects recovering from COVID-19, may be a consequence of any acute illness with fever. The hair loss observed many weeks after SARS-CoV-2 infection is consistent with telogen effluvium (TE), a condition characterized by sudden, non-scarring hair loss 2–3 months after a triggering event [26]. In the case of COVID-19‐associated TE, the insult was hypothesized to be able to induce an immediate release of hair follicles from the anagen phase and a switch to the catagen and subsequently telogen phase [27]. Noticeable hair loss, reported by approximately 20% of COVID-19 survivors [20], has been associated with a more severe course of illness. The number of subjects in our study, however, was too low to draw conclusions about this association. TE typically affects less than half of the scalp and lasts for approximately six months [26]; a hair that falls out is replaced by a new, growing hair. As HED patients, due to the lack of sweating ability [28], are expected to develop high fever in the process of a SARS-CoV-2 infection and, thus, might experience more significant physical and emotional stress than control subjects with COVID-19, the increased frequency of TE could have been anticipated. It may even be underestimated in our cohort because adolescents with HED often suffer from a profuse disease-related hair loss [29] and many male HED patients become baldheaded during the third or fourth decade of their life.

## Conclusions

HED appears to go along with an increased risk of post-acute consequences of a SARS-CoV-2 infection, such as chronic fatigue and hair loss. Particular attention to these issues may be required in adolescents and young adults, and preventive vaccination against COVID-19 should be recommended for all individuals affected by HED.

## Supplementary Information

Additional file 1: Figure S1. Analysis of SARS-CoV-2 spike-blocking antibodies in the sera of HED patients using the SUBA assay (Schuh et al., 2021). In brief, 96-well plates were coated with recombinant human ACE2. Spike-expressing Ramos cells (Rsp cells) were then allowed to attach to ACE2 in the absence or presence of sera containing spike-binding and -blocking antibodies. Bound Rsp cells were then fixed and stained with crystal violet. Crystal violet staining was quantified using a spectrophotometer. Data are presented as mean % Rsp cell binding of triplicates relative to control (Rsp cells in the absence of serum or blocking antibodies). A serum sample which reduced Rsp cell binding to less than 50% was defined as “serum with spike-blocking antibodies”.

## Data Availability

The datasets used and analyzed during the current study are available from the corresponding author on reasonable request.

## Abbreviations

COVID-19: Coronavirus disease of 2019
FEV1: Forced expiratory volume over 1 s
HED: Hypohidrotic ectodermal dysplasia
MEF 75: Maximal expiratory flow at 75% of the forced vital capacity
PEF: Peak flow
RT-PCR: Reverse transcription-polymerase chain reaction
SARS-CoV-2: Severe acute respiratory syndrome-coronavirus type 2
TE: Telogen effluvium
VC: Vital capacity
