# Supplementary material for: Increased risk of chronic fatigue and hair loss following COVID-19 in individuals with hypohidrotic ectodermal dysplasia
Source: Orphanet J Rare Dis. 2021 Sep 3;16:373. doi: 10.1186/s13023-021-02011-z (PMC8414461; doi:10.1186/s13023-021-02011-z)
Supplement: Supplementary file 1 — Analysis of SARS-CoV-2 spike-blocking antibodies in the sera of HED patients using the SUBA assay (Schuh et al., 2021). In brief, 96-well plates were coated with recombinant human ACE2. Spike-expressing Ramos cells (Rsp cells) were then allowed to attach to ACE2 in the absence or presence of sera containing spike-binding and -blocking antibodies. Bound Rsp cells were then fixed and stained with crystal violet. Crystal violet staining was quantified using a spectrophotometer. Data are presented as mean % Rsp cell binding of triplicates relative to control (Rsp cells in the absence of serum or blocking antibodies). A serum sample which reduced Rsp cell binding to less than 50% was defined as “serum with spike-blocking antibodies”. [file 13023_2021_2011_MOESM1_ESM.pdf]

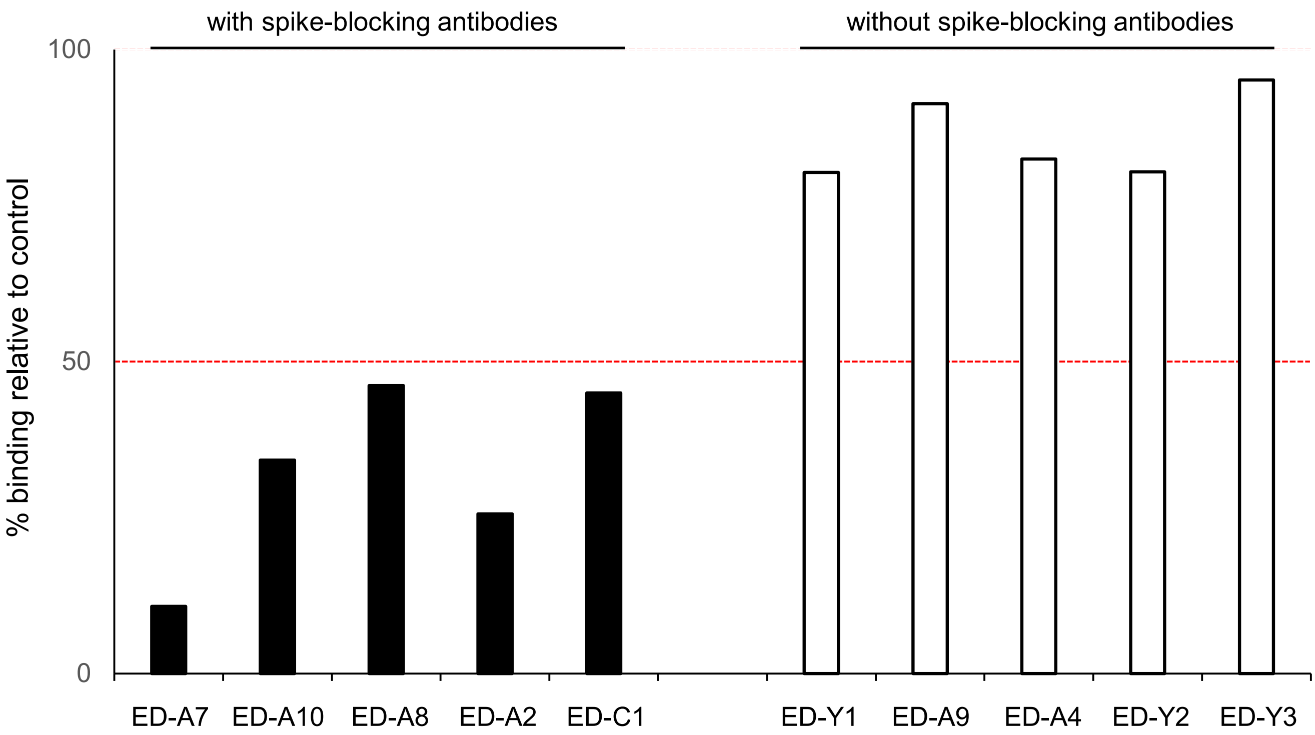

Supplementary figure: Analysis of SARS-CoV-2 spike-blocking antibodies in the sera of HED patients using the SUBA assay (Schuh et al., 2021). In brief, 96-well plates were coated with recombinant human ACE2. Spike-expressing Ramos cells (Rsp cells) were then allowed to attach to ACE2 in the absence or presence of sera containing spike-binding and -blocking antibodies. Bound Rsp cells were then fixed and stained with crystal violet. Crystal violet staining was quantified using a spectrophotometer. Data are presented as mean % Rsp cell binding of triplicates relative to control (Rsp cells in the absence of serum or blocking antibodies). A serum sample which reduced Rsp cell binding to less than 50% was defined as „serum with spike-blocking antibodies“.
